# Fragmented employment inclusion and lifespan health outcomes for autistic adults: systemic barriers and implications in China

**DOI:** 10.1186/s12916-026-05052-y

**Published:** 2026-07-07

**Authors:** Xi Wang, Xiaoli Guo

**Affiliations:** 1 Department of Sociology, Jilin University, No. 2699, Qian Jin Road, Changchun, China; 2 School of Economics and Management, Changchun University of Technology, No. 2055, Kuanping Road, Changchun, China

**Keywords:** paid absence, fragmented inclusion, autistic adults, employment, China, parents, parent-led organizations

## Abstract

**Background:**

Employment is a core social determinant of long-term health, well-being, and social inclusion across the autism lifespan, yet autistic adults face severe, globally pervasive employment inequities. Existing research has disproportionately focused on childhood diagnosis and intervention, with limited exploration of systemic barriers to substantive employment inclusion for autistic adults, particularly in non-Western family-led welfare contexts amid state welfare retrenchment. This study addresses this gap by examining how multi-stakeholder goal misalignment shapes fragmented employment inclusion for autistic adults and its implications for health equity.

**Methods:**

This study integrates Job Fit Theory and Regulatory Focus Theory to conduct an exploratory qualitative analysis, using maximum variation sampling to recruit 37 key stakeholders (parents, parents-led organizations, pre-employment training institutions, and employers) across four Chinese cities (Beijing, Guangzhou, Xi’an, and Changchun). Data were collected through 48 semi-structured interviews and long-term participant observation of sheltered workshops and cross-sector negotiation meetings, with thematic analysis applied for data coding. Autistic adults were not directly interviewed in this study because we focused on key interest-holders who directly shape their employment trajectories and daily work experiences.

**Results:**

Core findings reveal three critical forms of fragmented inclusion: parents’ collective prevention orientations narrow skill-demand matching to behavioral compliance, isolating autistic adults from mainstream labor markets; employers’ promotion-prevention conflicts result in symbolic hiring (“paid absence”—a practice where employers keep autistic adults on the payroll and pay them salaries but do not require them to attend work or perform meaningful tasks), maintaining formal employment status while undermining substantive inclusion; and these dynamics form a “proxy-driven fragmented inclusion paradox,” trapping job fit in a low-equilibrium state where short-term employment stability perpetuates capability deprivation.

**Conclusions:**

This study reveals how policies redistribute regulatory labor to families, exacerbating health inequities for autistic adults. These findings point toward a normative ethical framework that balances protection and autonomy and underscore that employment policies must empower autistic adults as active agents in their own employment trajectories. The findings offer critical insights for global autism employment policy and health equity practice for autistic adults.

**Supplementary Information:**

The online version contains supplementary material available at https://doi.org/10.1186/s12916-026-05052-y.

## Background

Employment is universally recognized as a foundational social determinant of long-term health, well-being, and social inclusion for autistic individuals across the lifespan [1, 2, 3, 4]. However, current research insufficiently addresses “dynamic job fit matching” across the full employment lifecycle of autistic individuals [5]. A particularly understudied phenomenon is fragmented inclusion—employment that meets some but not all of an autistic adult’s needs and interest-holders’ goals—especially in China contexts where family-led welfare provision fills gaps.

In China, a unique form of symbolic employment has emerged: employers hire autistic adults to comply with government-mandated disability employment quotas, yet exclude them from active work roles while continuing to pay salaries (a practice herein termed “paid absence”) [6, 7, 8, 9, 10]. This allows employers to avoid paying the disability employment security fund and fulfill corporate social responsibility (CSR) obligations without incurring workplace accommodation or behavioral support costs. This study addresses the core question: Under limited state support in China, how do goal differences between family proxies (parents and parent-led organizations) and employers shape fragmented inclusion employment for autistic adults? This question is rooted in realities of insufficient employment [7, 8], low expectations for full independence [9], and false employment [10]. Against this backdrop, understanding the mechanisms of fragmented employment inclusion and its impacts on lifespan health outcomes for autistic adults has become an urgent priority for research, policy, and practice, especially in non-Western settings like China, where pervasive and deeply entrenched social stigma [11] and low expectations for full independence [9] further undermine the effectiveness of existing employment policies and exacerbate systemic employment exclusion.

### Literature review

Most existing studies are based on medical and social models perspective [12], making it difficult to explain the core mechanism by which goal differences shape employment fit outcomes. Current research has confirmed that the employment dilemma of autistic individuals is related to heterogeneity [13], including difficulties in workplace communication and social interaction, repetitive behavioral patterns, and sensory sensitivities [14], alongside co-occurring neuropsychiatric conditions commonly associated with autism, such as attention-deficit/hyperactivity disorder (ADHD), anxiety, and depression, are frequently reported [15]. To improve employment integration, scholars have focused on structural factors, including parental involvement, vocational support, and transition services [16, 17], while employers often hesitate to hire or accommodate autistic individuals due to inadequate understanding [18]. Emerging international research highlights the critical role of co-workers and community members in fostering inclusive workplaces [5, 19, 20]. However, this pattern differs dramatically in China, a collectivist society where support is largely confined to immediate family [21, 22], and community involvement remains underutilized compared to Western contexts [23, 24]. This systemic absence of extra-familial social support forces families to assume full responsibility for their autistic adult children’s employment [25, 26]. The integration logic of family proxies’ and employers’ goals constitutes a multi-agent interaction perspective missing in existing research. Job Fit Theory [27] offers structural clarity for analyzing employment dilemmas, yet adopts a static, individual-focused lens, neglecting support systems and dynamic adjustments. This gap justifies integrating Job Fit Theory with Regulatory Focus Theory [28], which explains how stakeholder goals shape fit dynamics—forming the theoretical foundation of this study.

### Specificities of the Chinese context

The underemployment of autistic adults is a global crisis rooted in systemic failures of welfare states and labor markets [15, 25, 26], and China’s experience reflects both universal challenges and context-specific dynamics. While family-led support amid limited state backing is increasingly common worldwide [25, 26, 29, 30], China represents a special case of this global trend, where families have assumed near-complete responsibility for the employment and care of autistic adults.

China has over 10 million individuals with autism [31]. In earlier years, the employment rate among autistic individuals was less than 10% [7], though recent data suggest 40.4% of young autistic adults hold some form of (un) paid employment [8]. This improvement may be attributed to policy support from the Chinese government in the past decade [32, 33, 34, 35]. However, the profound employment quality deficits may have been overlooked: a significant proportion of these “employed” autistic individuals are in precarious, low-wage positions, and many are subject to “paid absence.”

Parental involvement in autistic employment support is a global phenomenon [25, 26, 29, 30]. In China, parents play a central role in the lives of their autistic adult children—even throughout their adult employment lifecycle [21]. Many parents have established organizations to provide pre-employment training and even sheltered employment venues [36, 37, 38]. This model arises from the absence of state-funded employment support [36, 38] and strong cultural norms of intergenerational family responsibility [11, 21, 39]. Collectivist values [40], low expectations for full independence [9], and pronounced social stigma in East Asia [41] further undermine Chinese existing disability employment policies [11].

Despite these dynamics, research on autistic employment in China rarely examines dynamic job fit from the perspective of goal differences between family proxies and employers—leaving critical gaps in understanding how structural factors shape the “fragmented inclusion” of autistic adults in employment.

This study adopts a qualitative interview method, focusing on three sub-questions: (a) How do family proxies’ prevention-focused strategies shape job-fit thresholds? (b) How do employers’ promotion-prevention tensions manifest in hiring practices? (c) Why does misaligned regulatory focus perpetuate fragmented inclusion? This study defines two core concepts: (1) Goal differences refer to inconsistencies in core goals and priority orientations between family proxies (i.e., parents and parent-led organizations) and employers involved in autistic adults’ employment. These goal differences are not inherently negative but can disrupt dynamic job fit when misaligned. (2) Fragmented inclusion—defined as employment that meets some but not all of an autistic adult’s needs and stakeholders’ goals—emerges as a key outcome of these goal differences. It lies between full inclusion (i.e., meaningful, supported employment that fosters autonomy and well-being; full independence through employment) and unemployment.

No studies have explored fragmented inclusion in the Chinese context, where family-led support and quota-driven hiring create unique dynamics. This study is the first to integrate Job Fit Theory and Regulatory Focus Theory to systematically explore how family proxies’ and employers’ goal differences influence the full employment lifecycle fit of autistic adults. While fragmented inclusion manifests uniquely in China, it is a global phenomenon affecting autistic adults in all countries with underdeveloped autistic employment systems [25, 26].

### Theoretical framework

We draw on two interrelated theories—Job Fit Theory and Regulatory Focus Theory—to reveal the essence of autistic adult employment gaps and the mechanisms underlying their persistence.

Job Fit Theory [27] provides structural clarity through its multi-dimensional matching framework (skill-demand, need-supply), but cannot explain why parents actively narrow fit dimensions or why employers accept formal fit (e.g., paid absence). It also overlooks informal support systems such as workplace mentors and community advocates, which act as critical bridges in Western contexts [15] but are weak in China where the entire burden falls on family members [42], who simultaneously serve as caregivers, advocates, vocational trainers, and on-site supervisors. This unique support structure. In contrast, Regulatory Focus Theory [28] has the advantage of explaining motivations: its prevention-promotion orientation model reveals the driving mechanisms behind parents’ collective actions (e.g., entrepreneurship to avoid risks) and employers’ mixed strategies (corporate social responsibility (CSR) image building and cost control). But it has structural flaws: the individual psychological orientation framework is unable to capture the collective regulatory levels derived from China’s institutional voids (e.g., parent organizations becoming independent regulatory subjects), nor does it connect the dynamics of matching outcomes. Integrating the two theories advances existing research in two critical ways. First, it captures the collective dimension of employment regulation that is invisible to single-theory perspectives, revealing how parent-led organizations dominate employment support through collective prevention strategies. Second, it explains stage-specific dynamic fit fractures across the full employment lifecycle, from distorted pre-employment training to post-employment symbolic fit. As research above reveals, families can emerge as the dominant, systemic ‘third support pole’ in contexts of extreme welfare retrenchment—a phenomenon that is increasingly observable globally but reaches its most developed form in China. This integration allows us to accurately diagnose the unique employment dilemmas of Chinese autistic adults and provide a transferable framework for studying occupational inequality under global welfare where parental involvement in autistic employment support.

## Methods

### Study design

This exploratory qualitative study adopts a demographic framework, aiming to answer the core question of fragmented inclusion employment for autistic adults during welfare transition. Qualitative methods are suitable for exploring “meaning and perspective” in population studies [43], while practical thematic analysis [44] helps identify patterns in family proxies-enterprises interactions.

As the purpose of the interview is to obtain diverse personal and professional perspectives and experiences, the maximum variation sampling method in purposive sampling was used to include a wide range of perspectives [45]. The design aligns with demographic dimensions: (1) Urban population stratification: Beijing (megacity, > 10 M), Guangzhou (metropolis, 5–10 M), and Xi’an/Changchun (large cities, 1–5 M) were selected to reflect how population size shapes the employment ecology (e.g., megacities face stronger CSR pressures but higher enterprise density). (2) Multi-agent data integration: Parents’ and parents-led organizations’ data illustrate “family proxies as demographic regulators”; enterprise data reflect “response to population policy quotas”; Pre-employment training institutions’ data bridge their interaction in different urban contexts. The data integration logic corresponds to the analysis framework of space policy.

### Inclusion criteria and recruitment

Participants were selected to represent key nodes in the “family proxies-enterprise” welfare network, with clear inclusion criteria for each group: (a) Parents or parent-led organization leaders must have provided continuous employment support for autistic adults for at least 2 years (e.g., participating in pre-employment training, job docking, or sheltered employment operations) to offer insights into families’ regulatory and supportive roles; (b) Enterprise representatives must have direct experience hiring autistic adults (e.g., participating in recruitment and management, negotiating or cooperating with parents or parent-led organizations) to capture corporate responses to the disability employment quota system—a core policy tool for disability employment—and fragmented inclusion; (c) Heads of pre-employment training institutions must have 4–7 years of intermediary experience (including both successful and unsuccessful cases) in connecting autistic adults with workplaces to provide insights into structural gaps in the welfare system.

Recruitment was primarily conducted via Disabled Persons’ Federations (DPFs), ensuring coverage of the “family proxies-enterprise” interaction nodes. The Disabled Persons’ Federation is a government-affiliated social organization in China that aims to protect the rights and interests of individuals with disabilities, coordinate disability-related support services, and connect individuals with disabilities with employers and service providers. As trusted intermediaries in local communities, DPFs assisted in identifying potential participants who met the inclusion criteria (e.g., parents of autistic adults, parent-led organizations, pre-employment training institutions, and employers who hire or are willing to recruit autistic adults) and distributing study information. This partnership ensured access to diverse and representative participants, particularly those from underserved communities who might be hesitant to engage directly with researchers. Snowball sampling was used to supplement DPFs’ referrals, with initial participants recommending other eligible individuals (e.g., employers in similar industries) to enhance sample diversity.

### Data collection

Data collection included the following components: (1) Participant observation of the environment in sheltered employment venues and the work status of autistic adults; and consultation meetings and field visits across two scenarios—negotiations between parent-led organizations and enterprises and enterprises collaborating with pre-employment training institutions—regarding job provision. Participant observation focused on agent-employer negotiations (e.g., job customization talks), revealing unspoken compromises (see Table 1); (2) 26 semi-structured interviews with leaders of pre-employment training institutions and the staff of the parent-led organizations, discussing goals, needs, priorities, strategies, and effectiveness (i.e., successes, and failures) across the full employment lifecycle, perceptions of enterprises’ attitudes toward autistic adults, and challenges in autistic adults’ employment; (3) 16 semi-structured interviews with 10 mothers of autistic adults, exploring how parents and parent-led organizations intervene, set goals, and develop plans throughout the employment lifecycle, the training strategies and implementation of pre-employment training institutions, whether these strategies enhance employment opportunities for autistic adults, and causes of employment failure; (4) 6 semi-structured interviews with representatives from three enterprises seeking to employ autistic adults, investigating conditions for successful employment, potential causes of failure, whether pre-employment training provided by parents, parent-led organizations, and institutions meets enterprise needs, perspectives on employment priorities for autistic adults, and employment barriers.

**Table 1:** Participation in Observation Details

Observation Scenario	Number of Observations	Total Observation Hours	Observation Location	Observation Focus
Negotiations between parents-led organizations and enterprises	8	28	Parents-led organization offices	Negotiations on job customization, salary, and cooperation terms; Unspoken compromises; CSR-related symbolic behaviors
Enterprises collaborating with pre-employment training institutions	3	14	Pre-employment training institutions	Training content alignment with enterprise needs; Autistic adults’ on-site work performance
Sheltered employment venues	1	8	Parent-led restaurants; Sheltered small supermarket; Sheltered manual workshop; Washing workshop	Autistic adults’ work status; supervision methods; interaction with colleagues

Semi-structured interviews were conducted with each participant, lasting 45–60 min, using a pre-developed interview guide focused on goal differences and employment experiences. Prior to each interview, participants provided oral informed consent for audio recording, and all interviews were recorded using a digital voice recorder to ensure accurate capture of responses. All semi-structured interviews were conducted in Mandarin Chinese, audio-recorded, and transcribed verbatim. Transcripts were then translated into English by a bilingual researcher with expertise in disability studies and qualitative health research. To ensure translation reliability, a second independent bilingual researcher back-translated a randomly selected subset (20%) of English transcripts into Mandarin Chinese, and the two bilingual researchers resolved discrepancies by referring to the original recordings to reach consensus. All translated transcripts were de-identified (removing names, addresses, and other identifying information) prior to data analysis. Semi-structured interviews were guided by a theory-driven interview outline (see Additional file 1: Interview data), which focused on goal differences, employment experiences, and perceived barriers across the full employment lifecycle. Participant observation targeted key interactional turning points (e.g., breakdowns in parent-employer negotiations) to capture unspoken compromises and unforeseen regulatory contradictions not evident in interview data alone. Data collection ceased when thematic saturation was achieved, defined as no new themes emerging from three consecutive interviews and observations.

### Data validation and analysis

We use the practical thematic analysis [44]. The analysis is done manually, and no software package is used. Analysis steps: (1) Transcribe the interview text, read, and write a memo by all authors, focusing on “goal orientation,” “strategy selection,” and “fit performance; (2) Second-level coding combined with the theoretical framework: Based on the theory of job fit, identify the fracture dimensions of “skill demand matching” and “demand supply matching,” and classify “prevention strategies” (such as behavioral training) and “promotion prevention conflict strategies” (such as paid absenteeism) based on the theory of adjustment points; (3) Through team discussions to reach a consensus on the topic, ensure that the coding results can directly respond to the research questions used to test the mechanism of “goal difference → strategy selection → fragmented inclusion employment.”

### Methodological rigor

Ensuring research rigor through multiple strategies: (1) Multi-subject data triaging: comparing the descriptions of the same interaction event by parents, parents-led organizations, employers, and pre-employment training institutions to verify the consistency of target bias; (2) Participant review: Provide feedback on the topic summary to two participants and correct any misunderstandings; (3) Theoretical consistency test: Ensure that the analysis process always revolves around the core dimensions of work fit and moderation theory, avoiding thematic deviations. The coherence of the research process and the matching of methods and problems meet the rigorous standards of qualitative research.

## Results

Three themes and six subthemes emerged to characterize stakeholders’ perspectives on prioritized planning for autistic adults in China. Pre-employment training provided by family proxies and proxy job-seeking on behalf of autistic adults represents a common trajectory in their employment journey. While family proxies believe their autistic adult children are capable of holding employment roles, enterprises are reluctant to bear the costs of behavioral modification and management of order disruptions caused by autistic adults. Employers’ “paid absence” strategy epitomizes a regulatory fit failure: prevention-focused cost avoidance negates enterprises’ promotion-focused claims, crystallizing in low-equilibrium job fit (i.e., fragmented inclusion). The demographic characteristics of participants are presented in Table 2.

Themes represent broader concepts (see Table 3).

**Table 2:** Demographic Characteristics of Participants

Participant Type	Number of Participants	City Distribution	Key Demographic Features
Parents of autistic adults	10 (all mothers)	Beijing (1), Guangzhou (1), Xi’an (4), Changchun (4)	All have long-term experience (2 years) supporting autistic adults’ employment; five are members of parents-led organizations
Parents-led organization leaders/executives	19	Beijing (1), Guangzhou (4), Xi’an (8), Changchun (6)	Founders or core managers of parent-led organizations; 16 have autistic adult children
Pre-employment trainers	5 (decision makers, teachers, executives)	Beijing (1), Guangzhou (1), Xi’an (1), Changchun (2)	4–7 years of intermediary experience in autistic pre-employment training and matching employment
Enterprise representatives	3 (decision representatives)	Xi’an (3)	Responsible for CSR and employee recruitment; have experience hiring autistic adults

**Table 3:** Core Theme, Sub-Code, and Supporting Quote

Core Theme	Sub-Code	Supporting Quote
Parental Collective Prevention as Regulatory Labor: Narrowing Job Fit “Safety Thresholds”	Dual Roles: Mothers as Caregivers and Pre-Employment Trainers: Prevention Orientations	“If inappropriate daily behaviors aren’t addressed, they’ll persist in the workplace. This saves costs, but my child disobeys me more easily because I’m their mother.”
Parental Collective Prevention as Regulatory Labor: Narrowing Job Fit “Safety Thresholds”	Collective Entrepreneurship for Sheltered Employment: Group Prevention Orientations	“Only parents truly understand and accept their adult children. We opened a restaurant where our kids work at the front desk, supervised by us. As long as the business survives, they have secure salaries.”
Perceived “Safe” in Autistic Traits: Simplifying Fit Dimensions through Prevention Orientations	Reliability in Repetitive Tasks: Suitability for Service Industries	“Our children are hardworking and non-complaining, ideal for physically intensive roles.”
Perceived “Safe” in Autistic Traits: Simplifying Fit Dimensions through Prevention Orientations	Adaptation to Low-Communication Roles: Proactive Abandonment of “Social Fit” via Prevention Orientations	“Most enterprises accept low-communication roles for autistic adults. We negotiate with employers to assign simple tasks, like car washing.”
Employer Hybrid Orientations and Symbolic Fit: Formalizing Fit Amid Promotion-Prevention Tension	Social Behavior Mismatches: Undermining “Social Fit” via Promotion-Prevention Conflicts	“I don’t mind a non-verbal food runner, but when an employee expresses affection by touching female colleagues, it risks sexual harassment complaints.”
Employer Hybrid Orientations and Symbolic Fit: Formalizing Fit Amid Promotion-Prevention Tension	Symbolic Employment (Paid Absence): “Low-Equilibrium Fit” Under Promotion-Prevention Conflicts	“We fulfill our social responsibility by paying salaries, but hiring dedicated trainers for a handful of employees is financially untenable.”

### Theme 1: parental collective prevention as regulatory labor: narrowing job fit “safety thresholds”

In the context of institutional voids in China, family proxies translate their prevention-focused orientation into collective “adjustment labor,” establishing a safety threshold for job fit by narrowing the scope of skill-demand alignment. This theme encapsulates participants’ insights across the entire employment lifecycle, shedding light on family proxies’ aspirations and the institutional-level planning shaped by the unique challenges of supporting autistic adults. While this collective prevention strategy sustains superficial “skill-demand” matching in the short term, it overlooks the alignment of “needs and resources”—including social needs—thereby laying the groundwork for long-term fractures in employment fit.

#### Dual roles: mothers as caregivers and pre-employment trainers—prevention orientations

For most participants, pre-employment training—geared toward aligning autistic adults’ behaviors with social norms and workplace expectations—stands as the first critical barrier to overcome. Parental correction of daily behavioral patterns serves as a fundamental prerequisite for formal vocational training. As one mother put it: “If inappropriate daily behaviors aren’t tackled upfront, they’ll carry over into the workplace.” Many parents choose to deliver this training on their own rather than enlist professional help, even amid challenges: “This helps cut down on expenses, but my child tends to defy me more simply because I’m their mother.” Despite such hurdles, parents’ willingness to take on training responsibilities remains widespread. Mothers’ dual roles illustrate how prevention orientations dominate strategic choices, and this dynamic reveals that “behavior-first” training sows the initial seeds of misalignment in needs-resource matching.

#### Collective entrepreneurship for sheltered employment: group-level prevention orientations

Expanding this adjustment to the group dimension, the collective prevention-focused orientation of parent-led organizations builds sheltered employment venues via collective entrepreneurship—essentially a form of customized skill-demand alignment that limits task scope to fend off workplace conflicts. Participants highlighted the pervasiveness of parent-led collective entrepreneurship as a viable employment pathway for autistic adults. As one mother noted: “Most service providers catering to autistic adults in China are founded by parents themselves—rehabilitation centers, group homes, special education facilities—and these are often places where their autistic adult children take on jobs.” This observation reflects a strong sense of initiative among families in supporting employment for autistic loved ones.

A parent from a parent-led restaurant in Changchun shared in detail: “We joined hands with eight other families raising autistic adults to launch this restaurant—each family puts in capital and takes turns overseeing daily operations. Our autistic children work at the front desk, handling basic order-taking and table cleaning, with us close by to step in if they run into communication problems with customers. The business only makes modest profits, but it secures our kids a steady salary and a space free from discrimination—something mainstream employers scarcely offer.”

This collective entrepreneurship model was found across all four sampled cities: parent-led enterprises or service-oriented ventures—including restaurants, car washes, cafes, handicraft workshops, and community-based care facilities—prioritize safety and stability over profit margins. These initiatives fill the institutional gap created by inadequate state welfare support and employers’ unwillingness to accommodate autistic adults. Such efforts mitigate behavioral mismatches with public expectations, cementing parent-led organizations as key players in sustaining employment continuity for autistic adults.

Furthermore, a pre-employment trainer explained: “Our car wash employs 12 autistic adults, with their parents on-site to supervise. We have no intention of competing with mainstream car wash services—we simply want a safe haven where our autistic adult children can work without being judged. This is similar to the parent-led programs I’ve learned about in Western countries. I’m a member of a national parents’ association, and I picked up this model at our annual conference. Here in China, though, there’s no government funding allocated to employment initiatives for autistic adults.”

### Theme 2: perceived “safety” in autistic traits: streamlining fit dimensions through prevention orientations

Family proxies frame autistic traits as a form of “safety”—a perspective rooted in their prevention-focused mindset—that streamlines the alignment dimensions within Job Fit Theory. At its core, this theme revolves around the prioritization strategies of family proxies, highlighting how they identify perceived strengths in autistic adults’ behavioral profiles and adopt tactics to mitigate future uncertainties. While this streamlining reduces the complexity of skill-demand alignment, it narrows the scope of fit dimensions and amplifies the misalignment with employers’ actual needs.

#### Reliability in repetitive tasks: suitability for service industries

Participants drew comparisons between autistic adults’ traits and those of other disabled groups, casting stereotyped behaviors as a unique asset. “Our autistic adult children are diligent and compliant—they won’t slack off or act out with their supervisors, making them well-suited for physically demanding roles,” noted one mother who also serves as a pre-employment training director. Participants acknowledged that this trait is not universal among autistic individuals: some excel at repetitive tasks, while others demonstrate strong problem-solving abilities (e.g., in handicraft design). However, employers predominantly prioritize repetitive task performance and compliance, positioning autistic adults—especially those with communication barriers—as ideal candidates for low-skill, high-repetition positions. This disconnect between the heterogeneity of autistic traits and employers’ rigid stereotypes restricts autistic adults’ access to higher-skill employment opportunities.

#### Adaptation to low-communication roles: proactive abandonment of “social fit” through prevention orientations.

“Social fit”—defined as adapting to workplace interpersonal interactions—is a core dimension of Job Fit Theory. Yet family proxies, guided by their prevention-focused mindset of avoiding social conflicts, proactively opt for low-communication roles, effectively sidelining this critical dimension. Pre-employment training institutions tailor role assignments to the varying communication capacities of autistic adults: those with severe verbal impairments are placed in roles requiring minimal interaction (e.g., car washing, general cleaning), while those with moderate communication skills are assigned to supported positions (e.g., assisted cashiering). As one pre-employment trainer shared: “We don’t rely on a one-size-fits-all model—each autistic adult has distinct strengths. But employers typically only embrace the lowest-communication roles, which forces us to curtail opportunities for those who can handle more engaging tasks.”

Another trainer elaborated: “Most enterprises will only accept autistic adults for low-communication roles. We negotiate with employers to assign simple tasks—like organizing merchandise or delivering drinking water—while leaving complex or high-risk duties to neurotypical employees.” The founder of a parent-led car wash offered a concrete example: “Our trained autistic staff handle basic vehicle rinsing; neurotypical team members take charge of high-value cars. Steering clear of premium vehicles and sales-related tasks cuts down on liability risks, even though it drives up operational costs.”

### Theme 3: employer hybrid orientations and symbolic fit: formalizing fit amid promotion-prevention tensions

Unlike family proxies’ singular prevention focus, employers navigate promotion-prevention tensions through tokenistic job fit. This theme highlights employers’ tendency to prioritize workplace stability over inclusive employment, laying bare the tensions between family proxies’ support goals and corporate risk aversion. Paid absence stands as the quintessential example of low-equilibrium job fit amid misaligned regulatory orientations. This form of symbolic fit upholds “employment status” in name but severes both skill-demand alignment and needs-resource matching in practice.

#### Social behavior mismatches: undermining “social fit” amid promotion-prevention tensions

Employers often grapple with conflicting promotion and prevention orientations when hiring autistic adults: their promotion focus inclines them to hire autistic individuals (to project an inclusive corporate image), yet their prevention mindset—centered on avoiding workplace disruptions—has no tolerance for behaviors that do not conform to social norms, directly eroding the “social inclusion” dimension of job fit. In practice, employers open to hiring autistic adults cited behavioral misalignment with social norms—not a lack of verbal communication—as the primary barrier to sustained employment. A restaurant manager shared: “I don’t mind a non-verbal food runner at all, but when an employee shows affection by touching female colleagues, it raises the risk of sexual harassment complaints. Incidents like this throw the entire workflow off track.”

A leader of a parent-led organization in Guangzhou added: “We frequently negotiate with employers to assign low-communication roles to autistic adults, but even then, behavioral issues lead to dismissals. One employer fired an autistic worker for humming loudly during shifts—something we tried to alert them to in advance, yet they refused to make any accommodations.” This conflict—between employers’ promotion goals (projecting an inclusive image) and prevention goals (upholding operational stability)—undermines the very foundation of social fit in employment.

#### Symbolic employment (paid absence): “low-equilibrium fit” amid promotion-prevention conflicts

When behavioral issues threaten workplace order, enterprises often resort to “paid absence”—continuing to pay salaries while excluding autistic employees from active work roles. All three interviewed employers acknowledged engaging in such symbolic hiring practices: keeping autistic adults on the payroll without requiring their attendance or assigning meaningful tasks.

A human resources (HR) director from a Xi’an-based company confirmed this: “We hired one autistic adult to meet the government’s disability employment quota. But their stereotyped behaviors—like repetitive movements that disrupt production lines—render on-the-job training cost-prohibitive. We keep them on the payroll without mandating their presence. This way, we comply with policy, steer clear of operational disruptions, and fulfill our corporate social responsibility (CSR) obligations. Behavioral support should fall to the state or families, not businesses. That’s why companies often opt to keep autistic adults on payroll without requiring attendance—to avoid the high costs of training.”

Another HR manager elaborated: “We hire one autistic adult to meet the quota—we pay them the minimum wage but don’t ask them to report to work. Training them to adapt to our workflow would be more expensive than paying the disability employment security fund, and we can’t guarantee other employees won’t complain about their behavior.”

Family proxies generally accept paid absence arrangements as a pragmatic compromise, prioritizing financial stability over meaningful employment. As one mother explained: “I know my daughter isn’t actually working, but at least she has a steady income and health insurance. If she quits this job, she’ll never find another one. It’s better than sitting at home doing nothing.” However, some parents expressed deep frustration: “My adult son just stays at home all day watching TV. He’s getting more and more withdrawn. This job isn’t helping him at all.” A parent-led organization leader noted: “We don’t like paid absence, but it’s the best we can get right now. Employers won’t hire our kids for real jobs, so we have to take what we can get.”

## Discussion

This study identified priority domains influencing employment outcomes for autistic adults by examining the perspectives of family proxies and employers, analyzing how alignment and misalignment between these stakeholders’ goals shape fragmented inclusion in autistic adults’ employment (see Table 4). Three critical insights emerged from the analysis: (1) Family proxies’ goal misalignment—rooted in prioritizing safety and routine—is associated with dependence on sheltered employment and reliance on family among autistic adults; (2) Employer hiring decisions are dictated by quota compliance rather than genuine job fit, and symbolic employment practices (i.e., paid absence) are associated with fragmented inclusion; (3) Pre-employment trainers act as intermediaries, striving to bridge goal gaps between parents and employers but are constrained by limited resources.

**Table 4:** Goal Differences → Fragmented Inclusion

Chain Link	Stakeholder	Core Goal/Attitude	Specific Behaviors	Impact on Inclusion
Goal Differences	Parents/Parents-led Organizations	Prevention orientation: Prioritize safety and stability over development	Establish sheltered workplaces; Focus on behavioral compliance training; Earning a salary takes priority over career development	Narrow employment options; isolate from mainstream workplaces
Goal Differences	Enterprises	Comply with the government’s policies and employment ratio; Promotion-prevention conflict: Pursue CSR image while controlling costs	Hire autistic adults for symbolic purposes; avoid extra support costs	Prioritize formal compliance over substantive inclusion
Don’t Expect Full Independence Through Employment	Families	Accept limited independence; Prioritize financial sustenance	Collective entrepreneurship; Priority for sheltered employment; Compromise on employment development; Tolerate restricted roles	Reinforce autistic adults’ dependency on family support
Don’t Expect Full Independence Through Employment	Pre-employment training institutions	Align with family goals; adapt to enterprise demands	Design low-skill training	Limit skill development for independent work
Paid Absence	Enterprises	Cost control and CSR compliance	Keep autistic adults on payroll without work duties; Avoid risks	Maintain only formal employment status
Paid Absence	Family proxies	Accept financial stability over meaningful work	Tolerate paid absence; Do not demand substantive work opportunities	Perpetuate fragmented inclusion employment; Avoid challenging systemic barriers
Fragmented Inclusion Employment	All Stakeholders	Uncoordinated goals and strategies	Lack of institutional coordination; No lifecycle support system; Lack of supporting meaningful, and supported employment that fosters autonomy and well-being	Entrench occupational inequality; Hinder mainstream integration

### Family proxies as secondary regulators and narrowed job fit dimensions

The first key finding is that family proxies have emerged as secondary regulators amid state welfare retrenchment. They translate individual prevention-focused orientations into collective regulatory labor—an observation that challenges the Western “state/market dual regulation” hypothesis [46] and offers a new analytical unit for global disability studies.

Job Fit Theory centers on dual alignment of “skill-demand” and “needs-resource” dimensions, yet this study finds that parents’ prevention orientations—rooted in prioritizing behavioral compliance—while fracturing the latter (e.g., vocational interests). Gaps in pre-employment training reflect a systemic imbalance: families shoulder prevention-related responsibilities, while employers shirk the costs associated with promotion-focused inclusion. Family proxies highlighted a critical lack of systematic pre-employment training, with most behavioral and daily living skill interventions delivered informally by parents themselves. This training focuses on behavioral norms, daily self-care capacities, basic communication skills, and independent living competencies—all foundational for workplace participation. For parent-led pre-employment training organizations, strategies are tailored to their children’s abilities: families of autistic adults with strong skill sets advocate for mainstream employment, while those with significant communication or behavioral challenges prioritize safety and stable income—even if it means accepting “paid absence” arrangements.

This dynamic locks in precarious job fit dynamics—family proxies optimize for “safe” yet narrowly defined skill-demand matches. In practice, this means creating work environments for autistic adults that minimize the physical and mental toll of job-related struggles. For employers, it translates to hiring autistic adults without disrupting daily operations. Yet this “behavior-first” approach overlooks the occupational needs of autistic adults [47]. Narrowed skill-demand alignment in the pre-employment phase inevitably leads to post-employment fit fractures [48]. In turn, it reinforces the “special population” label and hinders autistic adults’ integration into mainstream workplaces.

### Enterprises’ hybrid orientations and symbolic employment

The second key finding is that enterprises’ promotion-prevention tensions give rise to symbolic employment, trapping job fit in a low-equilibrium state. The near-total dearth of post-hire skill development aligns with emerging scholarship [48]: when autistic adults cannot keep pace with evolving job demands, they face either dismissal or placement in “paid absence” arrangements.

The Integrated Employment Success Tool offers employers a framework for workplace transformation strategies tailored to autistic individuals, but individual needs are context-dependent—shaped by both workplace specifics and environmental factors [49]. Notably, employers comply with disability employment quotas while dismissing post-hire training as an unjustified expense, a sentiment amplified by the absence of state-mandated support frameworks. This dynamic mirrors a global trend where corporate social responsibility (CSR) initiatives prioritize regulatory compliance over genuine inclusive employment. Employers commonly regard providing jobs to individuals with disabilities as a charitable act, one that merely fulfills CSR obligations [50]. For families, the burden of post-employment support falls disproportionately on caregivers, who often provide full-time unpaid accompaniment—perpetuating financial insecurity and limiting their own workforce participation.

In the absence of state coordination, enterprises’ prevention-focused priorities overshadow their promotion-oriented commitments, reducing “inclusion” to a mere box-ticking exercise for compliance.

### Parental entrepreneurship and the “symbolic employment paradox”: theoretical implications of collective prevention

The third key finding is that compromise interactions between family proxies and enterprises give rise to a fragmented inclusion trap for autistic adults’ employment. Family proxies and employers operate under disparate regulatory logics: one anchored in prevention-driven survival, the other in navigating trade-offs between promotion and prevention orientations. As one pre-employment trainer stressed: “The longest continuous employment tenure for the autistic adults we’ve trained is one year; most are sent back by enterprises after roughly six months—yet those enterprises still agree to keep paying their salaries.” This dynamic yields short-term stability in employment rates, but at the expense of long-term employment quality, aligning with the “disadvantage reproduction” principle in population studies.

It is precisely because autistic adults lack adequate support in mainstream employment initiatives [51] that family-proxy collective entrepreneurship has emerged as a dominant adaptive strategy. Families establish employment venues in the catering and service sectors to create sheltered employment opportunities. Across the four sampled cities, this family-proxy sheltered employment model prevailed: interview data indicate 80% of parents and parent-led organizations either operate or partner with ventures—including catering services, car washes, cafes, service-oriented ventures, or handicraft workshops.

This model may help address unemployment-related mental health risks for autistic adults [52] but hinges heavily on caregivers’ unpaid labor. Most parents work eight or more hours daily to manage operations, and autistic adults’ skill development is confined to basic tasks (e.g., cleaning, simple assembly). In these settings, family proxies do not anticipate autistic adults achieving full independence through employment [9]—a contrast to research highlighting autistic adults’ intrinsic motivations, where employment serves as a pathway to independence and psychological fulfillment, not merely economic subsistence [47].

Often, family proxies absorb the bulk of operational workloads [37] or hire additional staff, thereby sidelining skill development and reinforcing dependency. Critically, such arrangements muddy the lines between sheltered, supported, and competitive employment—categories essential for targeted policy design. The prioritization of short-term income stability over long-term skill progression runs counter to the United Nations Sustainable Development Goal (SDG) 8 mandate for inclusive, sustainable livelihoods [53, 54]. These hybrid sheltered models sustain minimal job fit while laying bare the costs of outsourced regulatory responsibilities. Chinese family proxies do not seek full employment independence for their adult children [9]—a cross-cultural parallel that underscores a universal challenge: when states and markets fail to support autistic employment, families step in, yet their efforts require institutional backing to avoid entrenching marginalization for autistic adults.

### Policy implications and global policy recommendations

While the Chinese government rolled out a series of consecutive policies between 2017 and 2025 to encourage employers to hire individuals with disabilities, the state of fragmented inclusive employment for autistic adults remains deeply problematic. In practice, family proxies and employers operate within disconnected regulatory spheres, creating a “low-equilibrium trap” for autistic adults’ employment. Our findings resonate with Holler’s [46] framework on how disability ideologies shape policy: “When we study the specific ways in which the concept of disability shapes employment policy, we find that a key influencing mechanism is related to the formation of ideological toolbox boundaries—operational conclusions drawn from the concepts of various elements of disability.” In China, familial support networks are not barriers to the employment of autistic adults but integral components of the ecosystem.

Notably, the central role of families in supporting autistic employment is not unique to China. In Western Europe, parents also play a critical role in creating employment opportunities for their autistic adult children, given the poor performance of disability employment providers [55, 56]. Such as, autism employment programmes are often based on a business case [19], and autism employment programmes are often limited to specific work that is thought to be a good fit for business objectives and autistic individuals [57]. In China, by contrast, parent-led organizations have organized themselves into a nationwide movement that has built a parallel welfare system for autistic individuals [58, 59, 60]. However, the goal differences between family proxies and employers shape fragmented inclusion employment for autistic adults. It is a global phenomenon that affects autistic adults in all countries with underdeveloped autistic employment systems. Our findings thus have global relevance: relying on families to fill welfare gaps creates sustainable burdens and perpetuates marginalization. Policies must move beyond simply encouraging family involvement: (1) Recognize and support family caregivers as employment advocates. While families should not be forced to assume regulatory responsibilities, they are often the most effective advocates for autistic adults. Policies should provide financial support, training, and resources to help families navigate the employment system without compromising their own well-being. (2) Governments should strengthen enforcement of disability employment quotas to ensure that hiring leads to meaningful work rather than “paid absence.” (3) Countries should invest in comprehensive, state-funded pre-employment training, job coaching, and post-employment support services for autistic adults.

### Normative ethical implications: balancing protection and autonomy

The findings of this study point toward a normative ethical framework that balances two imperatives: protection and autonomy. Protection reflects the well-intentioned collective prevention orientations of family proxies, who create sheltered employment and accept paid absence arrangements to shield autistic adults from workplace harm, social stigma, and the psychological toll of repeated employment failure. Autonomy, by contrast, reflects the genuine skill-demand matching and meaningful social integration that Job Fit Theory identifies as essential for employment quality, psychological fulfillment, and long-term health. This tension constitutes the core ethical dilemma revealed by this study: family proxies are not merely overprotective gatekeepers but rational actors navigating a system that offers no viable alternative to overprotection. We argue that resolving this dilemma requires moving beyond the binary of protection versus autonomy toward an empowerment model in which autistic adults are recognized as active agents in their own employment trajectories. Without such structural shifts, the proxy-driven fragmented inclusion paradox will persist, thereby perpetuating capability deprivation and health inequity.

### Limitations

This study is subject to two limitations: (1) The sample draws exclusively from four urban areas, with no inclusion of rural regions. Notably, welfare frameworks and employment landscapes in rural settings may differ markedly from urban contexts, so future studies should incorporate cross-regional comparisons to broaden generalizability. (2) Our findings are based solely on the perspectives of family proxies, employers, and pre-employment trainers, which may not accurately reflect the lived experiences of autistic adults. For example, while family proxies generally view paid absence as a pragmatic compromise, autistic adults may have very different views about the value of meaningful work, autonomy, and social inclusion. Future research must prioritize the perspectives of autistic adults. Future studies should explore how autistic adults experience different forms of employment (including sheltered employment, supported employment, and paid absence) and their impacts on health, well-being, and quality of life.

## Conclusions

This study demonstrates that tokenistic, fragmented inclusion may not deliver on the promise of improved quality of life and may perpetuate cycles of capability deprivation and health inequity for the autistic community. While these findings are grounded in the Chinese context, they may have broader relevance for countries where state support for autistic adult employment remains limited, and families bear significant caregiving responsibilities. The “proxy-driven fragmented inclusion paradox” offers a new framework for understanding why employment outcomes for autistic adults remain poor despite decades of policy efforts.

## Supplementary Information

Below is the link to the electronic supplementary material.

Additional file 1: Interview data. Semi-structured interview outline focused on goal differences, employment experiences, and perceived barriers across the full employment lifecycle.

## Data Availability

The data analyzed during the current study are not publicly available as they contain potentially sensitive participant information. Data sharing requests may be directed to the corresponding author, subject to ethics approval.

## Abbreviations

CSR: Corporate Social Responsibility
DPF: Disabled Persons’ Federation
ADHD: Attention-Deficit/Hyperactivity Disorder
SDG: Sustainable Development Goal
