# Supplementary material for: Fragmented employment inclusion and lifespan health outcomes for autistic adults: systemic barriers and implications in China
Source: BMC Med. 2026 Jul 7;24:537. doi: 10.1186/s12916-026-05052-y (PMC13637336; doi:10.1186/s12916-026-05052-y)
Supplement: Supplementary file 1 [file 12916_2026_5052_MOESM1_ESM.docx]

Additional file 1: Interview data

| ***The type of participating / respondents' background*** | ***content*** | ***Observation content*** |
| --- | --- | --- |
| Parents-led organizations / Decision makers and executives | 1. What are the core objectives of the organization (such as striving for employment opportunities for autistic groups, reducing the burden on parents, and promoting policy changes)? 2. How does the organization define "successful employment support"? Is this definition consistent with the individual goals of members' parents? 3. What specific services does the organization provide for its members (such as vocational training, enterprise docking, and sheltered workshops)? What is the design basis of these services? 4. How do organizations negotiate employment conditions with enterprises? Is there a standardized strategy (such as customizing posts and behavior evaluation)? 5. Does the organization prefer "sheltered employment" to "mainstream workplace integration"? What is the consideration behind it? 6. When cooperating with enterprises, does the organization lower the employment standard (such as accepting "symbolic employment") in order to achieve cooperation? 7. When the organizational goals conflict with the individual needs of some parents (for example, some parents want to integrate into the mainstream workplace, while the organization focuses on asylum), how to coordinate? 8. When resources are limited, how can organizations compromise between "covering more families" and "improving service quality"? 9. In the face of corporate CSR show behavior (such as hiring people with autism only for publicity), does the organization choose to cooperate? What's the reason? 10. What kinds of jobs are available for autistic adults in China? | 1. Observe whether the parents-led organization emphasizes "collective security," "risk avoidance" (prevention orientation), or "group development" and "social identity" (promotion orientation) in promotional materials or meetings. 2. Record the evaluation criteria of the parents-led organization for "employment success" (such as employment rate, job stability, and integration degree).   Strategy correlation   3. Observe whether the training content of the parents-led organization focuses on "behavior compliance" (such as observing discipline) rather than "professional skills" (such as communication skills). 4. Record the core demands (such as job customization, salary guarantee, and long-term cooperation) when the parents-led organization negotiates with the enterprise.   Compromise correlation   5. Observe the attitude of the parents-led organization when the enterprise proposes "symbolic employment" (such as tacit acceptance, open opposition, and limited cooperation). 6. Record the balance between "the interests of the majority of parents" and "a few special needs" in the internal decision-making of the parents-led organization. |
| Parents | 1. When you support your children's employment, what are your top priorities? 2. What elements do you think an "ideal job match" should contain? this 3. What specific measures have you taken to help your child get or keep a job? 4. How do you judge whether these strategies are effective? What is the basis? 5. When choosing an employment direction, do you prefer a "safe but limited job" or a "challenging but more open job"? Why? 6. When your expectations conflict with the requirements of the enterprise, how do you usually compromise? 7. Have you ever given up some important needs to keep their jobs? 8. What do you think of such corporate behaviors as "paid absence"? Does this meet your definition of "real employment"? 9. When working with other parents, do you need to compromise your personal goals with your collective goals? Give examples. 10. What do you think are the limitations of the current support strategy? If there were more resources, how would you adjust? 11. What kinds of jobs are available for autistic adults in China? | 1. Observe whether parents give priority to prevention targets such as "stability" and "conflict avoidance" or promotion targets such as "employment development" and "social integration" in employment consultation. 2. Record parents' attention to their children's professional interests.   Strategy correlation   3. Observe whether parents focus on "compliance" (such as observing rules) rather than "skill improvement" (such as post ability) in behavior training. 4. Record parents' communication strategies with enterprises (such as taking the initiative to make concessions, insisting on demands, and seeking third-party mediation).   Compromise correlation   5. Observe parents' reactions when the enterprise makes unreasonable demands (such as silent acceptance, euphemistic opposition, and forced compromise). 6. Record parents' trade-off behavior between "short-term stability" and "long-term development" (such as rejecting challenging positions to maintain the status quo). |
| Pre-employment training and matching employment institutions/Decision makers, teachers, and executives | 1. What are the core objectives of your organization in supporting the employment of autistic groups (such as promoting social inclusion, improving the quality of employment, and promoting policy improvement)? 2. Do these goals overlap or conflict with those of parent organizations and enterprises? 3. What methods do organizations use to connect autistic groups with enterprises (such as job evaluation, job adaptation training, and employer education)? 4. How does the organization balance the strategy of "short-term employment placement" and "long-term career development"? 5. When choosing a cooperative enterprise, are there clear screening criteria (such as enterprise sincerity and job quality)? 6. When the enterprise demands to lower the service standard (such as simplifying the training process) to control the cost, does the organization compromise? 7. When resources are limited, how can an organization choose between "serving more people" and "deeply supporting a few people"? 8. When cooperating with parents' organizations, do you adjust your service strategies (such as reducing the docking of mainstream workplaces) because of parents' preventive orientation (such as overprotection)? 9. Under what circumstances do you accept "symbolic employment" in exchange for resources? 10. How to coordinate when the professional judgment of the organization (such as a position that is not suitable for autistic patients) conflicts with the expectations of parents? 11. What kinds of jobs are available for autistic adults in China? | 1. Observe whether the pre-employment training institutions' mission statement or project design focuses on "systematic change" (promoting orientation) or "emergency support" (preventing orientation). 2. Record whether the pre-employment training institutions include "the subjective experience of autistic people" (such as job satisfaction) when evaluating the project's effect.   Strategy correlation   3. Observe whether the pre-employment training institution's training courses take into account "skill matching" (job fit theory) and "psychological adaptation" (such as coping with workplace pressure). 4. Record the cooperation mode between the pre-employment training institutions and the enterprise (such as long-term in-depth cooperation and short-term project cooperation).   Compromise correlation   5. Observe the coping strategies of the pre-employment training institutions when the enterprise only pursues a CSR image (such as refusing to cooperate, demanding substantive positions, and limited participation). 6. Record the reactions of the pre-employment training institutions when parents interfere (such as insisting on professional advice, partially compromising, and withdrawing from cooperation). |
| Enterprises/Decision makers | 1. What are the main objectives of your company in hiring employees with autism (such as fulfilling social responsibilities, filling job requirements, and enhancing the corporate image)? 2. What conditions (such as skills, behaviors, and team integration) do you think "ideal autism employee-job matching" should meet? 3. What measures has the enterprise taken to support employees with autism (such as job adjustment, training, and assistance from colleagues)? What is the design basis of these measures? 4. How to assess the effectiveness of these measures? 5. When recruiting, training, and managing autistic employees, what are the differences between them and ordinary employees? 6. When the needs of employees with autism conflict with the efficiency of the enterprise (such as needing extra support time and adjusting the workflow), how can the enterprise balance? 7. Does the company keep jobs (such as symbolic employment) that are not suitable for employees with autism in order to maintain an "inclusive image"? 8. When communicating with parents or organizations, has the enterprise made compromises in terms of job requirements and working environment? 9. When autistic employees have behavioral problems, do enterprises prefer to "adjust management style" or "terminate employment"? What is the decision-making basis? 10. Faced with the conflict between policy requirements (such as quotas for disabled employees) and actual operating costs, how can enterprises compromise? 11. What do you think of the phenomenon that employers prefer to pay salaries but don't allow their adult autistic children to work? 12. Among different types of disabilities, which type of disabled people do you prefer to hire? What kind of disabled people are more reluctant to hire? Why? | 1. Observe whether enterprises' propaganda emphasizes "social responsibility," "public image" (promoting orientation) or "job adaptation" and "efficiency improvement" (preventing orientation). 2. Record the core standards (such as skills, behaviors, plasticity) of enterprises when recruiting employees with autism.   Strategy class   3. Observe whether the enterprise provides "customized jobs" (such as simplifying processes and reducing social needs) or "standardized jobs" (consistent with ordinary employees) for employees with autism. 4. Record the training contents (such as skills training and corporate culture adaptation) of enterprises for employees with autism.   Compromise correlation   5. Observe the response of enterprises when autistic employees affect team efficiency (such as adjusting team structure, increasing support staff, and persuading employees). 6. Record whether there are symbolic inclusive behaviors such as "paid absence" and "nominal employment" in the enterprise, and the decision-making logic behind them. |
